# Barley *lys3* mutants are unique amongst shrunken-endosperm mutants in having abnormally large embryos

**DOI:** 10.1016/j.jcs.2018.04.013

**Published:** 2018-07

**Authors:** Frederick Cook, Aoife Hughes, Candida Nibau, Beata Orman-Ligeza, Nicole Schatlowski, Cristobal Uauy, Kay Trafford

**Affiliations:** a The John Innes Centre, Norwich Research Park, Colney Lane, Norwich, Norfolk NR4 7UH, UK; b The National Plant Phenomics Centre, Institute of Biological, Rural and Environmental Sciences (IBERS), Aberystwyth University, Gogerddan, Aberystwyth SY23 3EE, UK; c The National Institute of Agricultural Botany, Huntingdon Road, Cambridge, Cambridgeshire CB3 0LE, UK

**Keywords:** Barley, μCT scanning, Embryo, High lysine, *lys3*, Shrunken endosperm, Days after flowering, DAF, dry weight, DWT, giant embryo, GE, Normal embryo, NE, large embryo, LE, micro-computed tomography, μCT, shrunken endosperm, SE

## Abstract

Many shrunken endosperm mutants of barley (*Hordeum vulgare* L.) have been described and several of these are known to have lesions in starch biosynthesis genes. Here we confirm that one type of shrunken endosperm mutant, *lys3* (so called because it was first identified as a high-lysine mutant) has an additional phenotype: as well as shrunken endosperm it also has enlarged embryos. The *lys3* embryos have a dry weight that is 50–150% larger than normal. Observations of developing *lys3* embryos suggest that they undergo a form of premature germination and the mature *lys3* grains show reduced dormancy. In many respects, the phenotype of barley *lys3* is similar to that of rice *GIANT EMBRYO* mutants (affected in the *OsGE* gene). However, the barley orthologue of *OsGE* is located on a different chromosome from *Lys3*. Together these results suggest that the gene underlying *Lys3* is unlikely to encode a starch biosynthesis protein but rather a protein influencing grain development.

## 1 Introduction

Cereal grains consist of three genetically-distinct parts: endosperm, embryo and pericarp. Of these, the endosperm is by far the largest component and contains most of the starch in the grain. Starch accounts for most of the calorific value of cereal grains for human food and animal feed. However, the nutritive value of grains also depends on other minor grain components such as proteins, amino acids, oils, vitamins and minerals. In barley (*Hordeum vulagre* L.), as in most cereals, lysine content (as a free amino acid or as a constituent of proteins) is limiting for nutritive value (Galili and Amir, 2013). For this reason, at the Risø Institute in Denmark in the 1960-70s, mutagenized barley germplasm was screened for altered lysine content and a number of high-lysine (*lys*) mutants were identified (Doll et al., 1974; Doll, 1976). The *lys* mutant with the highest lysine content (44 percent higher than wildtype) was Risø1508 (*lys3a*) (Ingversen et al., 1973; Mossberg, 1969). This work led to a breeding programme using Risø1508 to improve the nutritional quality of barley, particularly for pig feed (Munck and Jespersen, 2009).

In addition to high-lysine content, some of these barley mutants have other phenotypes. For example, almost all Risø *lys* mutants have low-starch content and shrivelled (also described as shrunken) grains. Numerous other barley mutants (e.g. *sex*, *seg* and *notch*) have been identified primarily because of their shrunken endosperm but these also have high-lysine content. Thus, the *lys* mutants are a sub-group of a larger set of shrunken endosperm mutants that display high-lysine and low-endosperm-starch phenotypes. Several of the shrunken endosperm mutants of barley have been found to carry lesions in starch synthesis genes (Supplementary Table S1) and their study has increased our understanding of the role of the corresponding genes in controlling starch content and composition in barley and cereal grains generally.

In addition to the high-lysine/low-starch phenotypes, another phenotype observed in some of the shrunken endosperm mutants is abnormal embryos. The embryo abnormalities include larger-than-normal embryos (Risø mutants 1508 and 527) (Tallberg, 1977; Deggerdal et al., 1986), larger-than-normal cells in the scutellum of the embryo (Risø mutants 1508 and 16) (Olsen et al., 1984; Deggerdal et al., 1986) and high-starch content and/or larger-than-normal starch granules in the embryo (Risø mutants 7, 8, 16, 17, 527 and 1508) (Deggerdal et al., 1986; Olsen et al., 1984).

Several questions concerning the shrunken endosperm barley mutants remain to be answered. First, although in many of these mutants the gene responsible for the pleiotropic phenotype has been shown to be directly involved in starch synthesis in the endosperm (Table S1 and Morell et al., 2003), it is possible that other high-lysine, low-starch barley mutants carry lesions primarily affecting different metabolic or developmental pathways. For example, mutants with large embryos could have lesions in genes controlling embryo development akin to the large-embryo mutants of other cereal species. In rice (*Oryza sativa* L.), there is a GIANT EMBRYO (GE) mutant that, like some of the Risø barley mutants, also has enlarged scutellar cells, reduced endosperm size (Nagasawa et al., 2013; Yang et al., 2013; Xu et al., 2014) and nutritional enhancements including high lipid, high protein and high lysine (Zhang et al., 2005). The *GE* gene has been identified: it encodes a cytochrome P450 called CYP78A13. The orthologue of *GE* in maize is also believed to be responsible for a large-embryo phenotype, and an associated high-oil phenotype (Zhang et al., 2012).

Secondly, although embryo abnormalities have been described for several of the shrunken endosperm mutants, we do not know the extent to which these endosperm and embryo phenotypes are linked or the mechanisms underlying any such linkage. It is conceivable that mutations that lead to a reduction in the flux to starch in the endosperm may make more sugars available for embryo metabolism. This additional supply of carbon may lead to the synthesis of higher-than-normal amounts of storage compounds in the embryo and thus, to larger embryos.

Thirdly, some of the shrunken endosperm mutants of barley (Risø mutants 8, 13, 16, 17, 1508, sex7 and sex8) have reduced dormancy and a predisposition towards preharvest sprouting (Howard et al., 2012; Olsen et al., 1984). It is not known whether the reduced dormancy observed is related in some way to the abnormal embryos or independent of this phenotype.

To investigate these matters further, we studied the relationship between embryo and endosperm size in a collection of shrunken endosperm barley mutants (Supplementary Table S1). This led to a more detailed characterization of the large-embryo phenotype in the *lys3a* mutant, Risø1508 and three additional barley mutants with lesions at the *lys3* locus: Risø18 (*lys3b*), Risø19 (*lys3c*) and M1460 (*lys3d*) (Aastrup, 1983; Munck, 1992). We also considered whether the large embryo phenotype of *lys3* could be due to disruption of the barley orthologue of CYP78A13 (*HvGE*). Our results suggest that the *Lys3* gene is distinct from *HvGE* and that it represents a novel gene controlling embryo size and grain development in barley.

## 2 Materials and methods

### 2.1 Barley germplasm

The collection of low-starch barley mutants used here (Supplementary Table S1) was assembled from the following publicly-available germplasm collections: BBSRC Cereals Collection, JIC **(**Bomi, Glacier, Carlsberg II, Ingrid, Maythorpe, Golden Promise, Risø8, Risø1508, Pentlandfield Glacier, Risø13, Risø527, Notch2 and Risø16); USDA-ARS National Small Grains Collection, Idaho, USA (Bowman, NP113, Seg6, Sex8, Sex7, Risø86, Seg7 and Sex6); Nordic Gene Bank, Alnarp, Norway **(**Risø29 and Risø17); Tom Blake, Montana State University, USA (Nubet and Franubet); Birthe Møller Jespersen, University of Copenhagen, Denmark (Minerva, Risø18, Risø19, and M1460) and Alan Schulman, University of Helsinki, Finland **(**Shx). All of the mutants in the collection show seed-shrivelling under the control of a single recessive locus.

### 2.2 Plant growth

Barley was germinated by placing individual seeds directly into the wells of a 24-well seed-tray containing a peat and sand mix. Once the seeds had germinated and the seedlings were established (two to three weeks), each seedling was transplanted into a 1 L pot containing barley compost mix (375 L Levington M3 compost (Scotts Professional, Ipswich, UK), 100 L perlite, 200 L 4-mm grit, 1.6 kg Osmocote® (Scotts Professional, Ipswich, UK).

Barley was grown either in a glasshouse or a controlled environment room (CER). In the glasshouse in winter, additional lighting was provided by sodium lamps for 16 h per day and temperatures were maintained between 15 °C (night) and 20 °C (day). Barley grown in CER had conditions of 16 h light (15 °C) and 8 h (12 °C) darkness, 80 percent humidity and quantum irradiance of 300–350 μmol s^−1^ m^−2^ at ear height.

### 2.3 Harvesting developing grains

Time after flowering was used to determine the age of the grains. Anthesis occurs while the ear is enveloped in the flag leaf so the exact day of anthesis is difficult to determine without damaging the plant. Accordingly, flowering time was defined as the day on which the awns of the developing ear protruded more than 1 cm above the leaf sheath.

### 2.4 Preparation and weighing of embryos

To remove embryos from mature grains they were first imbibed overnight in sterile water at 4 °C in the dark before the embryos were removed, dried to constant weight and weighed (dry weight).

### 2.5 X-ray micro-computed tomography (μCT) scanning and photography

Mature, fully-dried primary spikes of barley were subjected to X-ray μCT scanning and analysis as described in Hughes et al. (2017). This non-destructive technique uses X-rays to inspect the interior of an object (in this case, a barley spike) and to create a virtual 3D model of the structure. Spikes from plants grown at the same time in the same glasshouse were used and at least three grains from the middle of six different spikes per genotype were scanned. Spikes were placed in scanning holders and loaded into the sample changing carousel of a mCT100 scanner (Scanco Medical, Switzerland) with the X-ray power set at 45kVp and 200µA with an integration time of 200ms. Attenuation of the X-rays as they cross the sample is detected as grey values. Output images were produced at a resolution of 7.4 μm/pixel (> than 2000 sections per scan). Longitudinal sections and (after axis transposition) transverse sections of grains were analysed using ImageJ (Schindelin et al., 2012). The slice where the embryo length was highest was selected for embryo area measurements using ImageJ measuring tools. Photographs of grains were taken with a digital camera (Olympus TG-3 Tough Cam Sports, OlympusCorporation, Tokyo) equipped with a ring light.

### 2.6 Carbohydrate extraction

Samples were extracted by grinding thoroughly for 90 s at 1500 rpm in 0.5 mL 0.77 M ice-cold perchloric acid in a Genogrinder® (SPEX CertiPrep, New Jersey, USA) using 1.5 mL polyvinyl tubes and stainless steel balls 3/8 inches in diameter. Following grinding, a further 1 mL of perchloric acid was added to the homogenate which was allowed to fully-acidify for 30 min on ice. The insoluble material, including starch, was separated from the soluble metabolites, including sugars, by centrifugation at 20 000 g for 10 min at 4 °C.

For embryo carbohydrate extraction, the supernatant was removed and the pellet re-suspended in 100 μL of sterile water. Following another centrifugation step as above, the second supernatant was added to the first.

The supernatants were used to measure glucose, fructose and sucrose. The pellets were assayed for starch as follows. Embryo samples: pellets were re-suspended in 250 μL of sterile water from which four representative aliquots were taken (two were digested and two were used as undigested controls). Endosperm samples: to remove excess soluble carbohydrate contaminants from the insoluble pellet, the pellet was washed by re-suspension in 1 mL of sterile water, followed by two re-suspensions in 1 mL of 80 percent ethanol solution. Finally, the pellet was re-suspended in 1 mL of sterile water from which four representative aliquots were taken for starch assay (two were digested and two were used as undigested controls).

### 2.7 Starch assays

Samples were autoclaved for 20 min at 120 °C to solubilise the starch. Starch was digested to glucose using α-amylase and amyloglucosidase (both from *Aspergillus oryzae,* manufactured by Megazyme Ltd., Co. Wicklow, Ireland) and the glucose measured enzymatically as described in Rösti et al. (2006). Coupling enzymes in the glucose assay, hexokinase and (NADP-dependant) glucose-6-phosphate dehydrogenase were manufactured by Roche Diagnostics (Basel, Switzerland). The reaction was monitored at 340 nm on either a spectrophotometer or a microtitre plate reader. Initial absorbance was recorded before the reaction was started by the addition of (NADP-dependant) glucose-6-phosphate dehydrogenase. The reaction was monitored until steady absorbance was reached. The difference between initial absorbance and final absorbance was used to calculate glucose in the samples. The mass of starch (g) was calculated as moles of glucose ×162. Undigested controls were included in the analysis to adjust for glucose in the pellet not from starch.

### 2.8 Glucose, fructose and sucrose assays

The supernatant from carbohydrate extraction was neutralised to pH 7.0 by addition of KOH/MES/KCl (2 M KOH, 0.4 M MES, 0.4 M KCl), centrifuged at 12 000 g to remove the insoluble potassium perchlorate and glucose, fructose and sucrose were measured enzymatically as described in Rösti et al. (2007). The sucrose was digested using invertase (β-fructosidase) manufactured by Megazyme (Co. Wicklow, Ireland). Fructose-6-phosphate was converted to glucose-6-phosphate using phosphoglucose isomerase (Roche Diagnostics, Basel, Switzerland). Free glucose and fructose contents were measured in undigested controls. Sucrose content was calculated by subtracting free glucose and fructose contents from those in the digested controls.

### 2.9 Protein extraction and assay

A single freeze-dried embryo (1.0–6.0 mg) was thoroughly ground, for 90 s at 1500 bpm, in 500 μL of sterile water in the Genogrinder® (SPEX CertiPrep, New Jersey, USA, using 1.5 mL polyvinyl tubes and stainless steel balls 3/8” in diameter). Then 500 μL of protein extraction buffer (0.2 M MOPS pH 7.2, 4% (w/v) SDS, 2 mM DTT) was added and proteins were allowed to dissolve for 30 min at room temperature with continuous rocking. Samples were then centrifuged for 5 min at 12 000 g before the supernatant was assayed using a BCA (bicinchoninic acid) protein assay kit (Thermo Fisher Scientific, Rockford Illinois, USA) according to the manufacturer's instructions and using BSA standards.

### 2.10 Determination of seed dormancy and seedling growth

To study dormancy and after-ripening, plants grown in controlled environmental conditions were allowed to dry for several weeks without watering prior to harvest. Grain from the primary tillers were stored at room temperature in the dark prior to germination trials. To assess dormancy, germination rate was measured each week for four weeks after harvest. Germination trials were as follows: grains were allowed to imbibe/germinate on moist filter paper at 17 °C in the dark. After five days, the number of grains with a visible radicle was scored.

Moisture content of grains during the after-ripening period was measured as follows: samples of 20 grains were weighed, incubated at 80 °C for >48 h and re-weighed. The loss of weight during incubation was taken to indicate moisture content.

Germination rate was also measured in grains more than 5 weeks after harvest, when the grains were assumed to be no longer dormant. Grains were allowed to imbibe/germinate on moist filter paper at 17 °C in the dark. The germination rate was measured at intervals for 22 h after imbibition. Germinated grains were scored when the radicle was first visible outside the hull. Root and shoot length were measured five days after imbibition.

### 2.11 DNA extraction

DNA was prepared from seedling leaves using a DNeasy Plant Mini Kit (QIAGEN) according to the manufacturer's instructions.

### 2.12 Identifying the chromosomal location of the barley orthologue of OsGE

PCR primers were designed to amplify the barley (cv. Betzes) *GE* orthologue and to discriminate against the wheat (cv. Mardler) orthologous homoeologues. The primers were *HvGE* forward 5′-AGGCGTACGTCGGTAACATC-3'and *HvGE* reverse 5′- TCGGTATGCTTGTAGAATGTAGAG-3’. All PCR reactions were performed using Phusion Hot Start II high-fidelity DNA polymerase (Thermo Scientific) according to the manufacturer's instructions. The PCR thermal cycler was programmed as follows: initial denaturing step of 2 min at 94 °C; 35 cycles of 30 s at 94 °C, annealing for 30 s at 45–60 °C and extension at 72 °C for 30 s kb^−1^; and final extension step of 72 °C for 10 min. PCR products were separated on a 1.5% agarose gel and visualised by ethidium-bromide staining under UV light.

## 3 Results

### 3.1 All four lys3 mutants have larger-than-normal embryos

It has been shown previously that Risø1508 (*lys3a*) has larger-than-normal embryos. To test whether the other *lys3* mutants also have large embryos, all four mutants and their wild-type controls were grown together and the mature grains were visually examined (Supplementary Fig. S1). This showed that the large-embryo phenotype was observed in Risø1508, Risø18, and M1460 mutants and in the grains of some, but not all of the Risø19 plants tested. Observations of Risø19 plants showed that there were two phenotypically-distinct types present: all had shrunken grains but only some had grains with large embryos (Supplementary Fig. S2). For further studies of Risø19, two pure-breeding lines were isolated and designated Risø19LE (large embryo) and Risø19NE (normal embryo). Measurements of grain and embryo weights confirmed that Risø1508, Risø18, M1460 and Risø19LE have large embryos compared to their wild-type controls (Table 1).

**Table 1:** The effects of *lys3* on grain and embryo size.

	Grain DW (mg)	Embryo DW (mg)	Embryo DW (% grain DW)
Bomi	53.29 ± 2.16	2.06 ± 0.05	3.9 ± 0.1%
Risø18	44.91 ± 2.18	3.14 ± 0.03	7.0 ± 0.3%
Risø19LE	41.68 ± 0.76	3.39 ± 0.12	8.1 ± 0.3%
Risø1508	45.79 ± 0.67	3.84 ± 0.13	8.4 ± 0.3%
Minerva	45.27 ± 1.83	1.65 ± 0.04	3.6 ± 0.1%
M1460	39.73 ± 1.41	4.59 ± 0.13	11.6 ± 0.1%

Twenty mature grains from the same plant were imbibed in water overnight and then the embryos were excised. The embryo and non-embryo samples were each pooled, oven-dried and weighed (DW = dry weight). Grain DW is the weight of the embryo sample plus that of the non-embryo sample. Values shown are per grain and are means ± SE for samples from three or four different plants. For all data shown, the values for the mutants were statistically significantly different from the values for their respective wild types (Student's *t*-tests; P ≤ 0.05). All plants were grown in the same glasshouse in the same year except that Minerva and M1460 were sown one month later than the other genotypes. Bomi is the wildtype parental control for *lys3* mutants Risø18, Risø19LE and Risø1508. Minerva is the wildtype parental control for M1460.

**Fig. 1:**
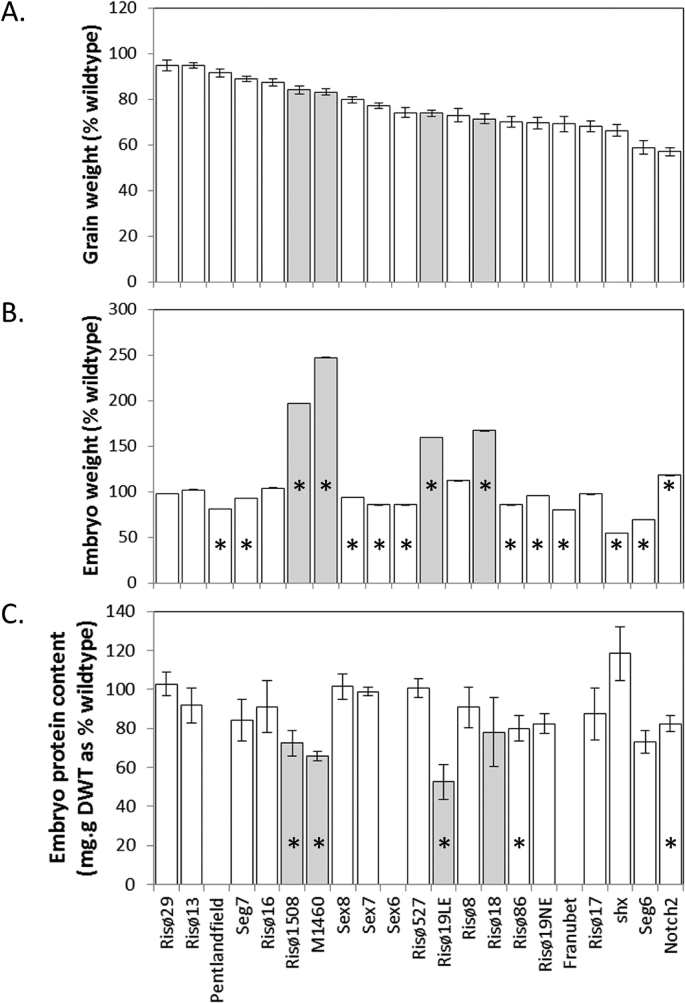
**Mature grain and embryo weights and embryo protein contents**. Samples of mature grains were collected and weighed (A). The embryos dissected from the grains were also weighed (B). Values (A, B) are means ± relative standard errors for 10 to 25 replicate grains from a minimum of two separate plants. Extracts of embryos were assayed for protein content (C). Each sample consisted of an individual embryo from a separate plant. Values (C) are means ± SD of measurements of three to five samples. Values (B, C) that are significantly different from control values (Student's *t*-test, p < 0.05) are indicated by an asterisk. *Lys3* mutants are shown in grey and all other mutants in white.

To test whether all of our *lys3* mutant stocks, particularly Risø19LE, carry lesions at the *Lys3* locus, Risø1508 was crossed with Risø18, M1460 and Risø19LE. F_1_ seed from these crosses was visually inspected and all F_1_ seed were found to have a large-embryo phenotype (data not shown). Given the previously reported recessive nature of the *lys3* mutants investigated here, this showed that lesions at the *Lys3* locus are almost certainly responsible for the large-embryo phenotype. The identity and origin of the Risø19NE grains is not known. The grain size of Risø19NE is lower than normal (Fig. 1A). Possibly the original Risø19 stock of grain had become contaminated with grain from another shrunken-endosperm mutant line. Alternatively, the original mutant line could have had lesions in both the *Lys3* locus and in another locus, and that these two mutations were not inherited together in subsequent generations.

### 3.2 A large-embryo phenotype was not observed for all shrunken endosperm barley mutants

We investigated whether shrunken endosperm barley mutants commonly have enlarged embryos by examining a selection of twenty shrunken endosperm mutants (Supplementary Table S1). As well as the *lys* mutants, there are shrunken and notched mutants that were originally selected according to endosperm shape. These include mutants in which the shrunken-endosperm phenotype is determined by the filial genotype (collectively known as *sex* mutants: shrunken endosperm xenia), and those in which it is determined by the maternal genotype (*seg* mutants: shrunken endosperm genetic). So far, the genes responsible for only five of the twenty mutant lines in Supplementary Table S1 have been identified. These all carry lesions in genes directly involved in starch biosynthesis (*Nst1* - plastidial ADPglucose transporter, *Agps1* – small subunit of ADPglucose pyrophosphorylase, *Isa1* – isoamylase, *Amo1* – starch synthase IIIa, *Flo6* – controlling starch granule size and number) (Supplementary Table S1).

Measurements showed that, as expected, all of the selected mutant lines had lower grain weights than their respective controls (Fig. 1A). Of the twenty mutant lines examined, ten had embryo weights that were low compared to their wild-type controls, five had embryo weights equivalent to wild-type and five had increased embryo weights (Fig. 1B). Of the five lines with increased embryo weights, four of these were *lys3* mutants. The embryo weights of the *lys3* mutants ranged from 160% of wild type for Risø19LE to 247% for M1460. The largest increase in embryo weight amongst the shrunken endosperm mutants other than *lys3* was 118% for Notch 2 (an isoamylase mutant), which has the smallest grain size of all the mutants studied. These data suggest that 1) most of the mutants with reduced grain size have both reduced endosperm size and reduced embryo size and 2) all four of the *lys3* mutants were clear outliers with respect to increased embryo weight. Thus, the large-embryo phenotype is not a common response to reduction in endosperm growth. It is however, a particular feature of mutants with lesions at the *Lys3* locus.

### 3.3 μCT scanning reveals lys3-dependent defects in both endosperm and embryo

**Fig. 2:**
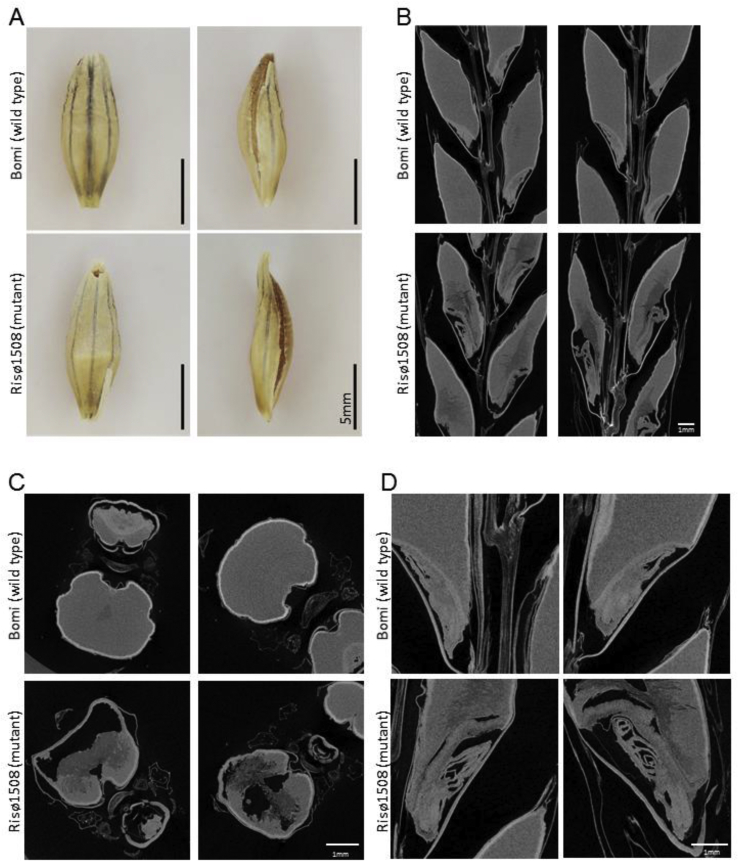
**Embryo and endosperm development in the wild type and *lys3a* mutant**. Three grains from the middle of six mature, fully-dried primary spikes per genotype were imaged by X-ray micro-computed tomography (μCT) scanning. Images were obtained as slice stacks and representative images of wild type (Bomi) and *lys3a* mutant (Risø1508) grains are shown. (A) Photographs of mature grains. (B–D) Representative images of sections through mature barley spikes obtained by μCT scanning. (B) Longitudinal sections through the scanned grains highlighting differences in endosperm structure. (C) Transverse sections of the scans shown in (B) highlighting the void space present in the mutant grains. (D) Enlarged images of the embryo region of grains shown in (B) illustrating that unlike wild-type embryos, the coleoptiles in mutant embryos were frequently expanded and the leaves within were well separated.

The grain and embryo phenotype of the *lys3a* mutants were studied further by μCT scanning (Fig. 2). This allowed access to the grain components in the context of the spike without dissection and specifically, it allowed us to examine the endosperm and embryo structures in more detail. As expected, the wild-type grains were seen to be fully-filled with continuous endosperm and the grains of Risø1508 were shrunken. Unexpectedly, void spaces were often observed in the endosperm of Risø1508 (Fig. 2B and C) and unlike the scutellum in the wildtype, the mutant scutellum was enlarged and was not in close contact with the endosperm (Fig. 2B and D). We also observed differences in endosperm density with the mutant grains showing a more irregular pattern of density, possibly related to altered endosperm development and/or composition (Fig. 2B and C).

In agreement with previous reports and with our measurements of embryo weights (Fig. 1B), the wild type and Risø1508 mutant differed in embryo size. Based on cross-sectional area measurements, Risø1508 mutant embryos were found to be bigger than wild type embryos (1.82 ± 0.18 mm^2^ and 1.31 ± 0.66 mm^2^ respectively; p < 0.05). Additionally, many of the Risø1508 mutant embryos were seen to have a distinct shape and texture (Fig. 2D). Whereas in wild-type embryos, the coleoptiles were always dense in texture and contained tightly-packed leaves, in Risø1508 embryos, the coleoptiles were frequently expanded and the leaves within were well separated. To quantify this effect, we scored grains for the embryo phenotype: 15 out of 23 Risø1508 embryos displayed expanded coleoptiles, whilst this was never observed in the wild type, Bomi (n = 18).

### 3.4 Embryos of lys3 are larger-than-normal from an early stage of development

**Fig. 3:**
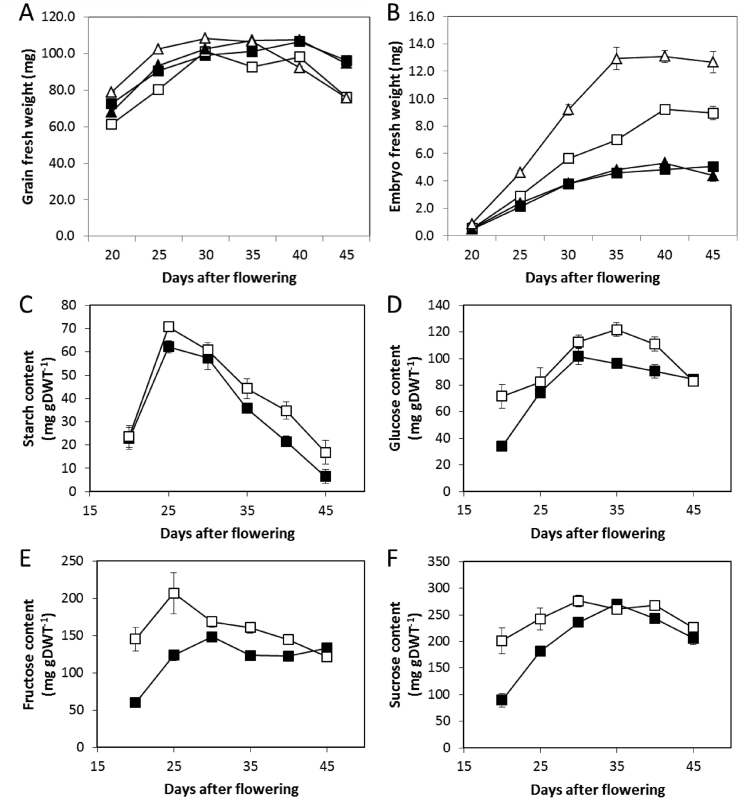
**Developing grain and embryo weights and embryo carbohydrate contents**. (A, B) Immature grains of the *lys3* mutants (Risø1508, white squares and M1460, white triangles) and their wild type controls (Bomi, black squares and Minerva, black triangles), respectively, were collected at various stages during grain development. Grains were weighed and the embryos excised and their fresh weight determined. Values are means ± SE for 25 replicate grains from 4 to 5 separate plants. (C to F) Immature wild type (Bomi, black squares) and *lys3* mutant (Risø1508, white squares) embryos were excised and dry weight (DWT) determined. Embryo samples were extracted and assayed for starch and soluble sugars. Each sample consisted of six to eight embryos and was from a separate plant. Values are means ± SE for 3 to 5 replicate samples.

To determine when during grain development the large-embryo phenotype becomes apparent, two of the *lys3* mutant lines were studied in greater detail (Fig. 3). The fresh weights of Risø1508 and M1460 grains were similar to those of their wild type controls, Bomi and Minerva, respectively (Fig. 3A). However, the weights of the mutant embryos differed from the wild-type controls throughout most of the developmental stages examined (Fig. 3B). In M1460, embryo fresh weight was significantly higher (Student's *t*-test; P < 0.00001) than that of the control at the earliest time-point examined: 20 days after flowering (DAF). In Risø1508, embryo weight was significantly higher than that of the control at 25 DAF (Student's *t*-test; P < 0.0004). Thus, embryo size is affected earlier and to a greater extent in M1460 than in Risø1508. As Risø1508 and M1460 have differing genetic backgrounds, it is not possible from these data alone to determine whether the differences in phenotype are due to variation between these two *lys3* alleles or to effects of genetic background. The ratio of fresh-to-dry embryo weight changed during development but this was not affected by the *lys3* mutations, showing that the increase in embryo fresh weight in the *lys3* mutants was accompanied by a proportional increase in embryo dry weight (data not shown).

### 3.5 The composition of lys3 embryos is different from wild-type

The carbohydrate and protein contents of developing embryos of Risø1508 and/or M1460, and their wild-type controls were measured. The starch content of Risø1508 embryos was slightly higher than that of the wild-type control, Bomi throughout development (Fig. 3C–F). The contents of glucose, fructose and sucrose were all higher in the mutant embryos than in the wild types, particularly at the earliest stages of development. Taken together, these results indicate that the carbohydrate content per gram dry weight is higher in Risø1508 embryos than in wild-type embryos.

The protein contents of mature embryos for all four *lys3* mutant lines were compared with those of the other shrunken-endosperm mutants and their respective wild-type controls (Fig. 1C). None of the mutants examined accumulated more protein in the embryo than their respective wild type. Protein content per gram dry weight was significantly lower than normal in the embryos of three of the four *lys3* mutants Risø1508 (28 percent lower), Risø19 (50 percent lower) and M1460 (36 percent lower), and in the isoamylase mutant, Notch 2 and the ADPglucose transporter mutant, Risø86.

### 3.6 The lys3 mutation leads to reduced dormancy and aberrant shoot emergence

**Fig. 4:**
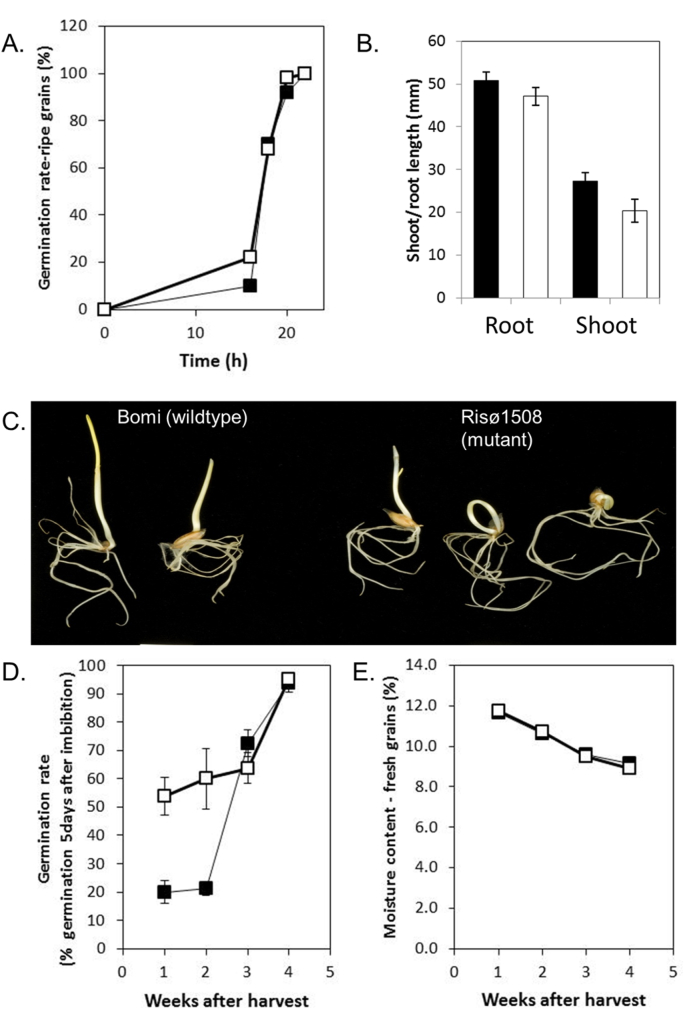
**Seed dormancy and seedling growth**. Dormancy and seedling growth in *lys3a* mutant, Risø1508 (white symbols/bars) was compared with that of wild type barley, Bomi (black symbols/bars). (A) Germination rate was measured in grains more than 5 weeks after harvest, when the grains were no longer dormant. The germination rate was measured at intervals for 22 h after imbibition. Values are percentages for samples of 50 grains. (B) Grains were germinated on moist filter paper at 17 °C in the dark. Root and shoot length were measured five days after imbibition. Values are means ± SE for 20 replicate grains. (C) Two examples of wild type seedlings, both showing normal shoot emergence, and three examples of Risø1508 seedlings, one showing normal shoot emergence and two showing impeded shoot emergence. Scale bar is 1 cm. (D) To assess dormancy, germination rate was measured each week for four weeks after harvest. Values are means ± SE for 4 replicate samples. (E) The moisture content of grains was measured each week for four weeks after harvest.

Germination and seedling growth were compared in the *lys3* mutant, Risø1508 and its wild-type control, Bomi (Fig. 4). To remove the potential effects of dormancy, grain that had been stored for more than three months was used. For stored (ripe) grains, there was no difference between Risø1508 and wild type in the time taken to germinate or in total viability (Fig. 4A). Both mutant and wild type showed 100 percent germination within 24 h.

Measurements of seedling growth (root and shoot length) at five days after imbibition showed that there was no statistically-significant difference in root length between mutant and wild type (Fig. 4B). However, in approximately half of the Risø1508 seedlings, shoot emergence was impeded due to the shoot apex becoming lodged within the enlarged scutellum (Fig. 4C). In the wild type, the elongating shoot travels between the hull and the endosperm and emerges at the distal end of the grain. In Risø1508, the emerging shoot often formed a ring, with the shoot apex lodged within the grain. In some instances, the shoot apex would turn through 180° and emerge from the proximal end of the grain. The Risø1508 shoots that emerged normally were not significantly different in length from wild-type shoots (Fig. 4B).

To compare dormancy, germination trials were carried out on mature grain immediately post-harvest (Fig. 4D). This showed that grain of the *lys3* mutant, Risø1508 was significantly less dormant than normal during the first two weeks after harvest. By four weeks after harvest, dormancy had been broken in both mutant and wild type. Moisture content can modify the imposition of dormancy during grain maturation and the breaking of dormancy during dry after-ripening. However, reduced dormancy in Risø1508 was not associated with altered grain moisture content (Fig. 4E). These results indicate that the *lys3* mutation leads to reduced dormancy but has no effect on grain viability or seedling growth once dormancy is broken.

### 3.7 Plant growth and development in lys3

Plants of the *lys3* mutant, Risø1508 and the wild type, Bomi were grown in controlled environmental conditions and their development was observed. The mutant and wild-type plants were indistinguishable in height throughout development. The growth rate in both decreased markedly around anthesis which occurred at 72 days after germination (data not shown). At maturity, a range of whole-plant metrics and yield components were measured (Table 2). Overall, the mutant and wild type plants were largely indistinguishable. Only three measurements were statistically significantly different between the mutant and wild type. In the mutant, the peduncle (the stem section subtending the spike) was on average, 2.7 cm shorter; the spikes were 8% shorter and the mean grain weight was 10% lower than in the wild type.

**Table 2:** Growth metrics and yield components.

	Feature	Bomi	Risø1508	*p* value
Spike				
	Spike length (cm)	10.3 ± 0.2	9.5 ± 0.2	0.007
	Number of grains per spike	26.4 ± 0.5	26.9 ± 0.3	0.46
	Grain weight (mg.grain-^1^)	66.5 ± 1.3	59.8 ± 1.1	<0.001
Whole plant				
	Number of tillers per plant	22.8 ± 1.6	25.5 ± 2.2	0.34
	Total weight of spikes (g.plant^−1^)	40.2 ± 2.4	39.7 ± 2.7	0.90
	Straw height (cm)	92.2 ± 1.6	88.3 ± 1.1	0.059
	Peduncle length (cm)	33.6 ± 0.4	30.9 ± 0.4	<0.001
	Total grain yield (g.plant^−1^)	32.7 ± 2.0	32.6 ± 2.1	0.97

Values are means ± SE of measurements made on 10 plants per genotype grown under controlled environmental conditions. Spike measurement did not include awns. Average grain weight was calculated from the weight of 100 grains per plant. Straw height was measured from the base of the stem to the top of the tallest peduncle. Peduncle length was measured from the first node below the spike to the base of the spike in three tillers per plant. Probability (*p*) values were calculated using Student's *t*-tests.

### 3.8 The barley orthologue of the rice giant embryo gene, OsGE, does not co-locate with Lys3

Identification of the gene underlying *Lys3* may allow separation of the favourable (nutritional enhancements) and unfavourable (yield depression) traits. This would potentially allow more effective utilisation of the *lys3* trait in barley and in other cereals. As a first step towards gene identification, we considered whether the *Lys3* gene might be orthologous to the gene responsible for the GIANT EMBRYO (GE) mutant phenotype in rice.

According to *Ensembl*Plants (http://plants.ensembl.org), the barley orthologue of the rice *Giant Embryo* (*GE*) gene (LOC_Os07g41240/ Os07g0603700) is HORVU2Hr1G032890. Thus, from the latest genome sequence information for barley, *HvGE* is located on chromosome 2H whereas *Lys3* is genetically mapped on chromosome 5H (Karlsson, 1977; Jensen, 1979; and our own unpublished results). To confirm the location of *HvGE* experimentally, we designed PCR primers specific for *HvGE* that did not amplify the orthologous wheat homoeologues and compared DNA extracted from wheat-barley addition lines (Supplementary Fig. S3). This confirmed that *HvGE* lies on the short arm of chromosome 2H.

## 4 Discussion

We have shown that all four mutants with lesions in the *Lys3* locus have large embryos and that most other shrunken-endosperm mutants do not have large embryos. Thus, it is possible to conclude that the increase in embryo size in *lys3* mutants is not a direct result of reduced starch biosynthesis in the endosperm. In fact, the majority of shrunken-endosperm mutants had a concomitant reduction in both grain weight and embryo weight. Additionally, for *lys3*, we found no evidence that a reduction in starch biosynthesis in the endosperm leads to an increase in protein biosynthesis in the embryo (Fig. 1) although there was an increase in starch and sugar content (Fig. 3).

The effects of mutations at the *Lys3* locus appear to be largely confined to the inflorescence and the reproductive tissues suggesting that *Lys3* expression is required during the development of these tissues. We showed that *Lys3* is required for normal flowering stem (peduncle) elongation, normal spike elongation as well as normal grain development, dormancy and germination. *Lys3* appears not to be required for normal vegetative growth although a reduction in straw height in Risø1508 compared to Bomi was described previously (Olsen, 1978).

As well as a reduction in dormancy in *lys3* grains, we observed an alteration in the appearance of the mature embryos using μCT scanning. Compared to the wild-type embryos, the mutant coleoptiles were frequently larger and contained leaves that were well-separated from each other. Together this suggests that in *lys3* grains, the normal dormancy mechanism is broken and that the mutant grains may undergo a form of premature germination, as suggested by Olsen et al. (1984). Phytohormones (gibberellins, ABA and ethylene) have been suggested to play a role in dormancy induction and breakage (Iglesias-Fernández et al., 2011) and hormonal abnormalities in Risø1508 have been described previously. Risø1508 exhibits a peak in gibberellin-like activity higher and later in development than the wild-type, Bomi and in Risø1508, auxin content is low (one tenth of the maximum content in Bomi) throughout development (Mounla et al., 1980). However, the increase in embryo weight from early stages of grain development points to an excess of cell division and/or growth. The effects of *lys3* could therefore be due to loss of a cell cycle inhibitor early in grain development or to a defect in the dormancy pathway, or both.

A predisposition towards premature germination appears to be a common feature of barley mutants with reduced endosperm starch content (Howard et al., 2012), and can be induced in normal barley by certain environmental conditions. However, the premature germination displayed by *lys3* mutants appears to be particularly severe. The extent of embryo enlargement observed in *lys3* mutants sets them apart from other shrunken endosperm mutants, including those with mutations directly affecting starch biosynthesis in the endosperm. Whilst it is possible that the reduced post-harvest dormancy observed in Risø1508 may be, at least in part, due to reduced induction of primary dormancy as a result of reduced starch accumulation in *lys3* mutants, it seems likely, given the abnormal phytohormone levels associated with this mutation, that *lys3* has a direct effect on overall grain development, rather than a primary effect restricted to starch synthesis. These ideas are also supported by the observation that the endosperm of *lys3* mutants is irregular and often contains void spaces, indicating that the choreography of development is impaired.

Identification of the *Lys3* gene will be required to determine its mechanism of action and whether or not it acts together with *HvGE* to control embryo size perhaps via regulation of phytohormone levels. Efforts to identify the *Lys3* gene by genetic mapping are currently underway in our lab.

## Declarations of interest

None.
