# Supplementary material for: Barley lys3 mutants are unique amongst shrunken-endosperm mutants in having abnormally large embryos
Source: J Cereal Sci. 2018 Jul;82:16–24. doi: 10.1016/j.jcs.2018.04.013 (PMC6142819; doi:10.1016/j.jcs.2018.04.013)
Supplement: Multimedia component 1 [file mmc1.docx]

**Supplementary Table S1.** **Barley germplasm used in this study.**

| **Protein** | **Line** | **Gene or locus** | **Parent line** | **Mutagen** | **Selection criterion** | **References** |
| --- | --- | --- | --- | --- | --- | --- |
| ADP-glucose transporter | Risø13 | *Nst1* | Bomi | EMS | High lysine | (Doll,1976; Patron *et al.*, 2004) |
|  | Risø29 |  | Carlsberg II | EMS | High lysine | (Doll,1972; Patron *et al.*, 2004) |
|  | Risø86 |  | Carlsberg II | EMS | High lysine | (Doll,1972; Patron *et al.*, 2004) |
|  | Sex7 |  | Bowman | Induced | Shrunken grain | (Franckowiak,1994) |
| AGPase SSU | Risø16 | *Agps1* | Bomi | Fast neutrons | High lysine | (Doll,1976; Johnson *et al.*, 2003) |
| Iso-amylase 1 | Risø17 | *Isa1* | Bomi | Fast neutrons | High lysine | (Doll,1976; Burton *et al.*, 2002) |
|  | Notch 2 |  | NP113 | EMS | High lysine | (Sekhara *et al*.,1976; Burton *et al*., 2002) |
| Unknown | Risø8 | *Lys4* | Bomi | EMS | High lysine | (Doll,1976) |
| Starch synthase IIIa | Pentlandfield Glacier | *Amo1 / SSIIIa* | Glacier | Spontaneous | High amylose | (Merritt,1967; Li *et al.*, 2011) |
| Unknown | Seg6 | *Seg6* | Ingrid | Spontaneous | Shrunken grain | (Ullrich and Eslick,1978) |
| Unknown | Seg7 | *Seg7* | Ingrid | Spontaneous | Shrunken grain | (Ullrich and Eslick,1978) |
| Unknown | Sex8 | *Sex8* | Bowman | Induced | Shrunken grain | (Franckowiak,1994) |
| Floury endosperm 6 | Franubet | *HvFlo6* | Nubet | Chemical | Aberrant starch | (DeHass *et al.*,1983; Saito *et al*., 2017) |
| Unknown | Risø1508 | *Lys3a* | Bomi | Ethylenimine | High lysine | (Tallberg,1973) |
| Unknown | Risø18 | *Lys3b* | Bomi | Na azide | Unknown | (Munck,1992) |
| Unknown | Risø19 | *Lys3c* | Bomi | Na azide | Unknown | (Munck,1992) |
| Unknown | M1460 | *Lys3d* | Minerva | Na azide | Low β-glucan | (Aastrup,1983) |
| Unknown | Risø527 | *Lys6* | Bomi | γ-rays | High-lysine | (Doll,1976) |
| Unknown | Shx | *Shx* | Bomi | Spontaneous | Shrunken grain | (Schulman and Ahokas,1990) |

**Supplementary Figure S1.** **Effects of Risø19LE and Risø19NE on grain and embryo size.**

Observations of Risø19 plants showed that there were two phenotypically-distinct types present: all had shrunken grains but only some had grains with large embryos. Two pure-breeding lines were isolated and designated Risø19LE (large embryo) and Risø19NE (normal embryo).

**A)** Photographs of front and side views of grains**.** The scale bar is 5 mm.

**B)** Comparison of mature grain and embryo weights. Each sample consisted of 10 grains from the middle of the primary tiller. Grains were weighed and then embryos excised and weighed. Values are means ± SE for samples from three separate plants per genotype. The difference between values denoted by the same lowercase letter is statistically significantly different (P<0.05; Student’s *t*-test).


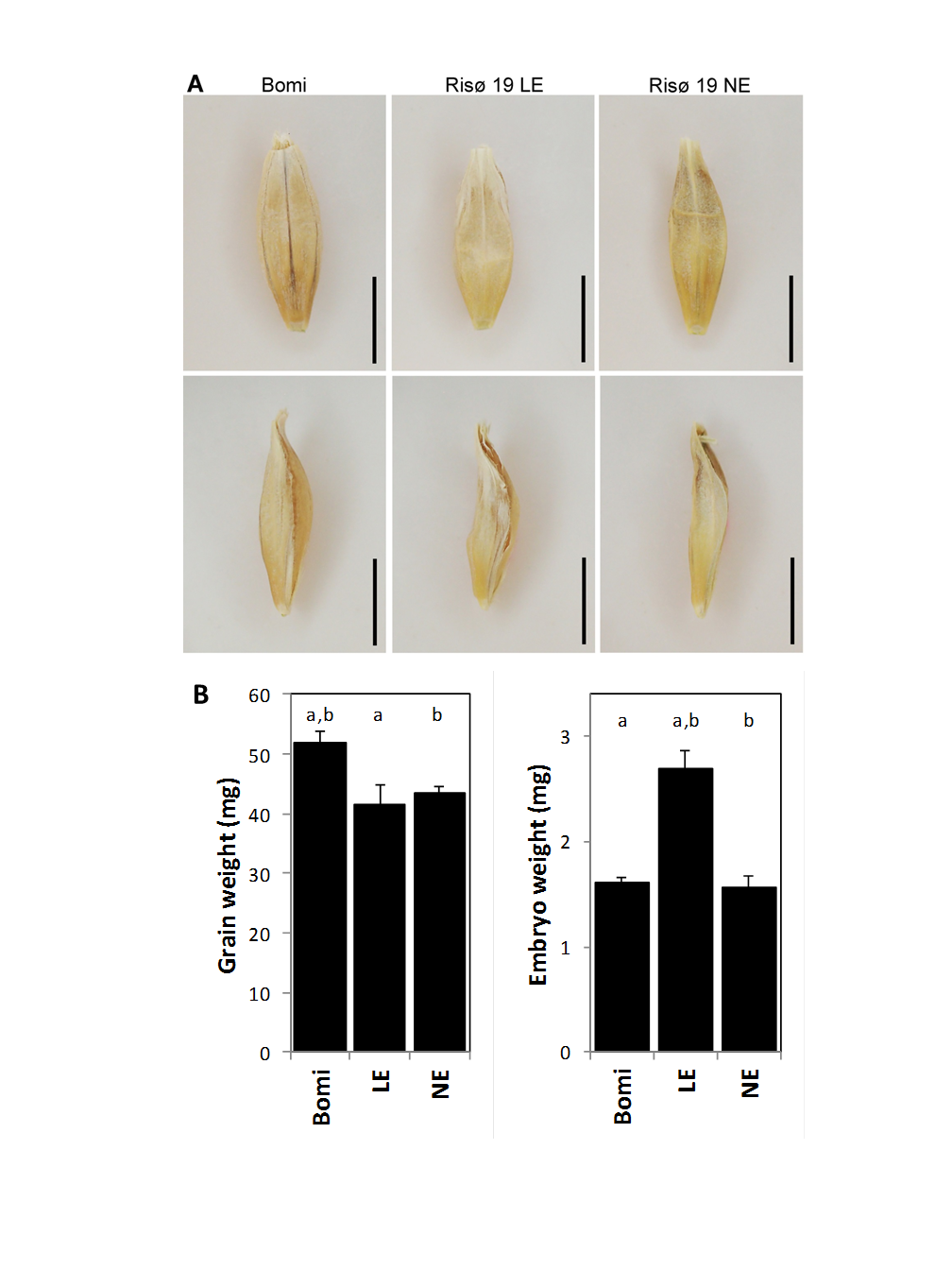


**Supplementary Figure S2.** **The effects of *lys3* mutations on barley grain morphology.**

Photographs of front and side views of the *lys3* mutant grains and their wild-type controls. Bomi is the wild-type control for Risø18, Risø19, and Risø1508 grains, whereas Minerva is the wild-type control for M-1460 grains**.** Bars = 5 mm.

**
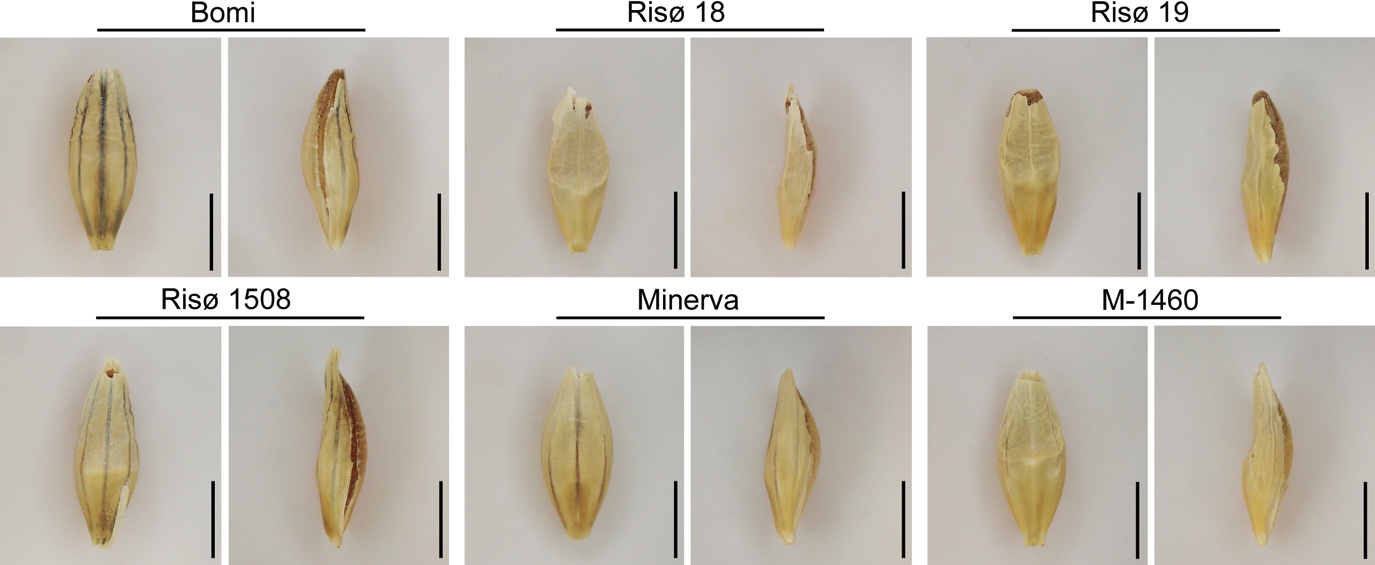
**

**Supplementary Figure S3.** **Identification of the chromosomal location of *HvGE*.**

Agarose gels showing ethidium bromide-stained PCR products are shown. The primers used were designed to amplify the barley orthologue of the rice giant embryo gene, *HvGE* but not to amplify the wheat *GE* orthologues.

**A.** The specificity of the primers was tested using barley (cv. Betzes) and wheat (cv. Mardler) genomic DNA as template.

**B.** *HvGE-*specific primers used to amplify genomic DNA from wheat-barley chromosome addition lines containing barley chromosomes 2H to 7H. No amplification was seen with addition lines 3H, 4H, 5H, 6H or 7H (not shown). A PCR product was observed with addition line 2H, and with a ditelosomic line containing the short arm of 2H (2HS) but not with a line containing the long arm of 2H (2HL).

**A.**

**B.**

Barley Wheat


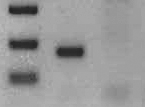


250 bp **-**

100 bp **-**

500 bp **-**


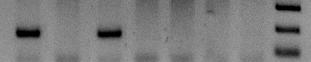


2H 2HL 2HS

100 bp -

250 bp -

500 bp -
